# Unguided Species Delimitation Using DNA Sequence Data from Multiple Loci

**DOI:** 10.1093/molbev/msu279

**Published:** 2014-10-01

**Authors:** Ziheng Yang, Bruce Rannala

**Affiliations:** ^1^Beijing Institute of Genomics, Chinese Academy of Sciences, Beijing, China; ^2^Department of Genetics, Evolution and Environment, University College London, London, United Kingdom; ^3^Department of Evolution & Ecology, University of California, Davis

**Keywords:** Bayesian species delimitation, species tree, multispecies coalescent, reversible-jump MCMC, guide tree, nearest-neighbor interchange

## Abstract

A method was developed for simultaneous Bayesian inference of species delimitation and species phylogeny using the multispecies coalescent model. The method eliminates the need for a user-specified guide tree in species delimitation and incorporates phylogenetic uncertainty in a Bayesian framework. The nearest-neighbor interchange algorithm was adapted to propose changes to the species tree, with the gene trees for multiple loci altered in the proposal to avoid conflicts with the newly proposed species tree. We also modify our previous scheme for specifying priors for species delimitation models to construct joint priors for models of species delimitation and species phylogeny. As in our earlier method, the modified algorithm integrates over gene trees, taking account of the uncertainty of gene tree topology and branch lengths given the sequence data. We conducted a simulation study to examine the statistical properties of the method using six populations (two sequences each) and a true number of three species, with values of divergence times and ancestral population sizes that are realistic for recently diverged species. The results suggest that the method tends to be conservative with high posterior probabilities being a confident indicator of species status. Simulation results also indicate that the power of the method to delimit species increases with an increase of the divergence times in the species tree, and with an increased number of gene loci. Reanalyses of two data sets of cavefish and coast horned lizards suggest considerable phylogenetic uncertainty even though the data are informative about species delimitation. We discuss the impact of the prior on models of species delimitation and species phylogeny and of the prior on population size parameters (*θ*) on Bayesian species delimitation.

## Introduction

Genetic sequence data have gained importance in delimiting species in recent years and several inference methods have been proposed for this purpose (reviewed in Fujita et al. 2012; Yang 2014). As noted by De Queiroz (2007), it is possible and indeed important to distinguish species delimitation from species definition or concept. Species concepts are often linked to particular mechanisms of achieving and maintaining genetic isolation between incipient species (e.g., the existence of complete reproductive isolation in the Biological Species Concept) but a useful delimitation method should not be wedded to any particular mechanism of isolation; instead it should be based on detecting the ultimate outcome of speciation—genetic isolation on an evolutionary timescale. If species are viewed as independently evolving metapopulations (De Queiroz 2007), it is reasonable to expect the genetic data to fit a species tree with the gene tree distributions described using the multispecies coalescent model (Rannala and Yang 2003). Coalescent-aware species delimitation methods have advanced considerably in recent years, with applications to many different taxonomic groups, such as lizards (Leaché and Fujita 2010), snakes (Ruane et al. 2014), and fungi (Lumbsch and Leavitt 2011).

A general approach to investigating the evolutionary and genetic structure of a group of related organisms using multilocus genetic sequence data can be envisioned as follows: 1) Assign individuals to populations whose members currently interbreed, 2) determine whether the populations are genetically isolated on an evolutionary timescale and are thus putative species, and 3) determine the phylogenetic history relating the delimited species (Wiens 2007; Yang and Rannala 2010). Currently, those three steps (population assignment, species delimitation, and phylogenetic inference) are usually carried out as separate procedures. However, all of them rely on similar information in the sequence data. Furthermore, errors and uncertainties in one (upstream) analysis may affect another (downstream) analysis (Leaché and Fujita 2010; Olave et al. 2014). Thus, a joint analysis should be optimal in maximizing the power and reliability of the inferences.

Several methods exist that are aimed at such a joint inference (Pons et al. 2006; O’Meara 2010; Ence and Carstens 2011). However, they involve simplifications and heuristics that lack rigorous statistical justifications (Yang 2014, p. 351). They all make use of estimated gene trees (topologies and branch lengths) but ignore uncertainties in the estimates. Sequence data from closely related species lack phylogenetic information and individual gene trees typically involve substantial sampling errors. This uncertainty is an important source of inaccuracy for both species delimitation and species tree inference (Leaché and Rannala 2011; Camargo et al. 2012). Some methods are applicable to data of only a single locus (Pons et al. 2006). More recently, tests of delimitation using Bayes factors have been proposed (Grummer et al. 2013; Leaché et al. 2014). These methods appear promising, although they suffer from the numerical difficulty of accurately calculating marginal likelihoods and can only test a limited number of prespecified hypotheses regarding species delimitations.

The Bayesian species-delimitation method of Yang and Rannala (2010) and Rannala and Yang (2013) has a number of advantages over heuristic methods (Fujita et al. 2012; Yang 2014). It is based on a sound statistical methology, and uses the multispecies coalescent prior (Rannala and Yang 2003) to accommodate the uncertainties in the gene trees. However, the implementation of Yang and Rannala (2010) relies on a user-specified “guide tree,” which completely specifies the topology of the species tree for the finest division of populations into species that is considered biologically plausible. The method then examines the support for various delimitation models that result from collapsing internal nodes in the guide tree (a collapsed node means that the descendent populations of the node constitute one single species). The requirement for a guide tree is a weakness of this approach. A grossly wrong guide tree, as generated by random permutations of populations, for example, may cause the method to oversplit (Leaché and Fujita 2010; Olave et al. 2014), although errors in estimated guide trees (as inferred from analyzing a fast-evolving mitochondrial locus or using a species-tree estimation method) do not appear to be a significant cause of spurious delimitations (Zhang et al. 2014). Even so, the data may contain much information about species status but little information about species phylogeny (see below). It is thus preferable to avoid the need for a guide tree so that phylogenetic uncertainty is accounted for in the calculation of the posterior probabilities of delimitations. Joint inference also reduces the burden of prior data analysis for the end-user. Eliminating the need for a guide tree should be particularly helpful for species identification through “DNA barcoding” (Dowton et al. 2014) in which case a guide tree and population assignments for one or more individuals to be identified are often conspicuously lacking. Thus, a practical and computationally feasible approach for joint assignment, delimitation, and species tree inference that takes account of major sources of uncertainty in the genetic sequence data is needed.

Here, we develop a new Bayesian inference procedure that jointly infers species delimitation and species phylogeny, and provides an initial solution to the problem of individual assignment to species as well. The problem is highly challenging as it spans the gulfs between population genetics, phylogenetics, and taxonomy. A major difficulty is the combinatorial explosion in the number of possible models of species delimitation and species phylogeny, and the resulting computational complexity. Nonetheless, a joint analysis using Bayesian computation based on Markov chain Monte Carlo (MCMC) appears feasible for moderate sample sizes (of individuals and populations). We extend our program BPP (for Bayesian Phylogenetics and Phylogeography) (Yang and Rannala 2010; Rannala and Yang 2013) to allow this joint inference. A novel MCMC proposal based on the nearest-neighbor interchange (NNI) algorithm for rooted trees is developed here to change the species tree topology, eliminating the need for a user-specified guide tree. The gene trees for multiple loci are altered in the proposal to avoid conflicts with the newly proposed species tree. We also modify our previous scheme for specifying priors for species delimitation models to construct joint priors for models of species delimitation and species phylogeny. As in our earlier method, our modified algorithm integrates over gene trees, taking account of the uncertainty of gene tree topology and branch lengths given the sequence data. We use simulations to examine the statistical performance of the method for different numbers of loci and species divergence times. We reanalyze two real data sets from cavefish (Niemiller et al. 2012) and coast horned lizards (Leaché et al. 2009) to illustrate the new method and to examine the relative information content in the data concerning species delimitation and species phylogeny.

## Theory

### NNI Algorithm to Modify the Species Tree Topology

We distinguish between a “population” and a “species.” Several populations may be grouped into one single species, but one population may never be split into two species. We use the terminology of Yang and Rannala (2010) and refer to a fully resolved phylogeny for the populations as a guide tree. Note however that the guide tree changes in the MCMC algorithm in this study. Internal nodes on a guide tree may be collapsed, generating fully specified models of species delimitation and species phylogeny. We use the NNI algorithm for rooted trees to propose changes to the species tree, with the number of species and the assignments of individuals to species fixed. As the current and new models during the NNI step involve the same number of parameters, there is no need for rjMCMC and we use MCMC. A second rjMCMC move in the algorithm proposes changes to species delimitations (by joining and splitting nodes in the current guide tree) and is essentially the same as that of Rannala and Yang (2013).

**Fig. 1.**
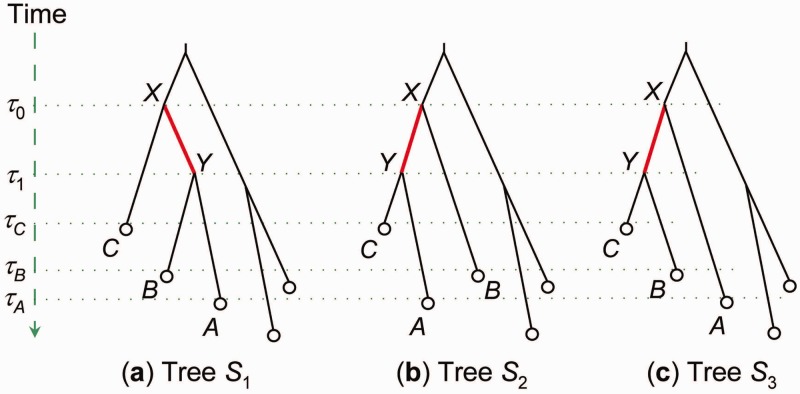
NNI on a rooted species tree. Each internal branch (say, *X-Y*) defines three possible trees relating three nodes *A*, *B*, and *C*. Given the current tree *S*_1_, the algorithm moves to one of the other two trees, *S*_2_ and *S*_3_, chosen at random.

Here, we describe the details of the NNI proposal. With equal probability we choose one of the internal branches on the species tree, say, *X-Y*. A branch is also referred to by the node it leads to, so that branch *X-Y* is also branch *Y*. The internal branch defines relationships among three nodes or subtrees: *A*, *B*, and *C*. The NNI move allows one to move from the current species tree *S*_1_: ((A,B),C) to one of two alternative species trees, *S*_2_: ((*C*, *A*), *B*) and *S*_3_: ((*B*, *C*), *A*) (fig. 1). Suppose the chosen species tree is *S*_2_. This is generated by pruning one of the branches *A* or *B*, chosen with equal probability (let it be *A*), and regrafting it onto branch *C*. We keep the ages of nodes on the species tree (ages *τ*_0_, *τ*_1_, *τ_A_*, *τ_B_*, and *τ_C_* for nodes *X*, *Y*, *A*, *B*, and *C*, respectively) unchanged during the move. The move is possible only if $\tau_{C}<\tau_{1}$; otherwise, it is disallowed.

**Fig. 2.**
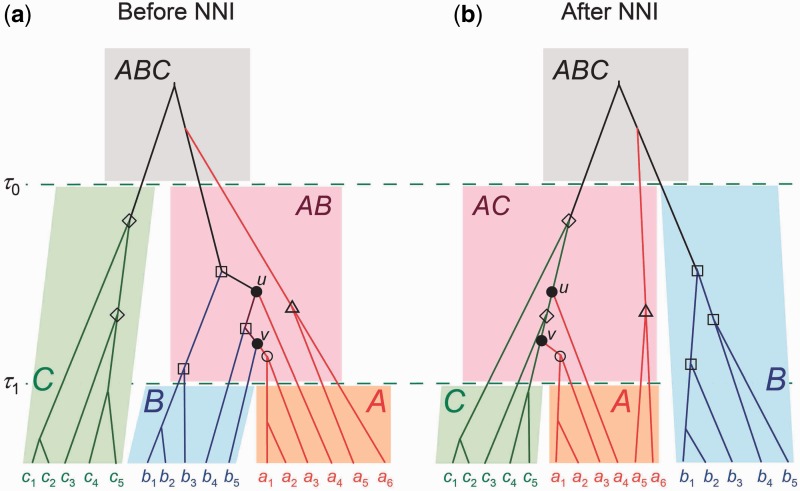
Some nodes on the gene tree are modified when the NNI algorithm is used to change species tree *S*_1_ to *S*_2_ in figure 1, that is, to prune species *A* and regraft it to branch *C*. A moved node (marked with •) lies in species *AB* and has exactly one daughter node with descendents in species *A* only. This, together with the subtree represented by the daughter node with descendents in species *A* only, is pruned and regrafted to a random contemporary branch in species *C*. In addition, four other kinds of “affected” nodes have their population IDs changed. They all have ages in the interval $\left(\tau_{1},\tau_{0}\right)$ and reside in either species *AB* or *C*. Any node marked with ○ or △ has descendents in species *A* only and changes its population ID from *AB* to *AC*. Any node marked with ⋄ is in species *C* and changes its population ID from *C* to *AC*. Any node marked with □ is in species *AB* with each of the two daughter nodes having descendents in species *B*, and changes its population ID from *AB* to *B*.

When we prune branch *A* and regraft it to branch *C* on the guide tree *S*_1_, we also move certain nodes on the gene trees to avoid conflicts. A gene-tree node that is moved this way, called a “moved node,” is defined as a node of age $\tau_{1}<t<\tau_{0}$ that lies in population *AB* and that has exactly one daughter node with descendents in population *A* only (e.g., nodes *u* and *v* marked with • in fig. 2). Every such node is moved together with species *A* and reattached onto a randomly chosen gene-tree branch that exists at time *t* in population *C*. We do not change the ages of any nodes on the gene tree during the move. Every node on the gene tree has a population identification (ID) uniquely specifying the population the node resides in. A moved node has its population ID changed from *AB* to *AC*. This pruning and regrafting move incurs a factor in the proposal ratio as the number of branches for attaching the moved node may differ between the source and target populations. Let $n_{C}(t)$ be the number of branches on the gene tree in population *C* at time *t*, and $n_{B}(t)$ be the number of branches in population *B* at time *t* in the reverse move (which prunes off branch *A* and reattaches it onto branch *B* in species tree *S*_2_ of fig. 1*b*). The moved node then incurs the factor $n_{C}/n_{B}$ in the proposal ratio. In the example of figure 2, we have $n_{C}=n_{B}=2$ for moved node *u* and $n_{C}=n_{B}=3$ for moved node *v*.

Besides the moved nodes, the NNI move on the species tree also affects four other kinds of nodes on the gene trees. These are called “affected” nodes. They reside in either populations *C* or *AB* and have ages in the interval $\left(\tau_{1},\tau_{0}\right)$, as illustrated in figure 2. However, for those nodes, the only change is to their population IDs, as their ages and topological relationships are not changed. Note that a change of population IDs does not incur any factor in the proposal ratio.

We note that if a locus lacks any sequences from population C and there exist moved nodes on the gene tree for the locus as defined above, our NNI move will be impossible as there will not exist any branch onto which to reattach the moved nodes. In such a case, the move is disallowed. Moves will become possible when the species tree (*τ*s) or the gene tree change in the MCMC so that there are no affected nodes for the locus. Note that our algorithm allows some populations to be entirely missing at some loci and also allows multiple sequences from the same population at any locus. The only requirement is that there must be at least two sequences at each locus.

### Combining NNI with rjMCMC

**Fig. 3.**
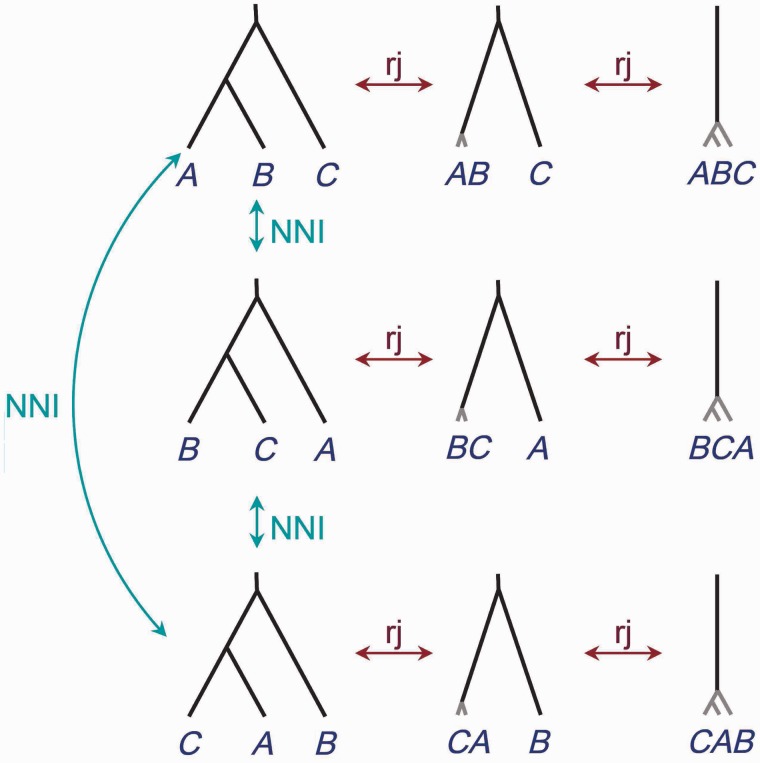
Models of species delimitation and species phylogeny for three populations *A*, *B,* and *C*. Models on the same row correspond to different species delimitation models given the same guide tree, formed by collapsing internal nodes on the guide tree (represented by short gray branches). The one-species model is represented three times, and there are nine models in our MCMC algorithm even though there are only seven biologically distinct models. The two priors constructed in this article assign equal probabilities ($\frac{1}{9}$) to the nine models. An NNI algorithm is used to move between the guide trees, whereas rjMCMC is used to move between species-delimitation models.

**Fig. 4.**
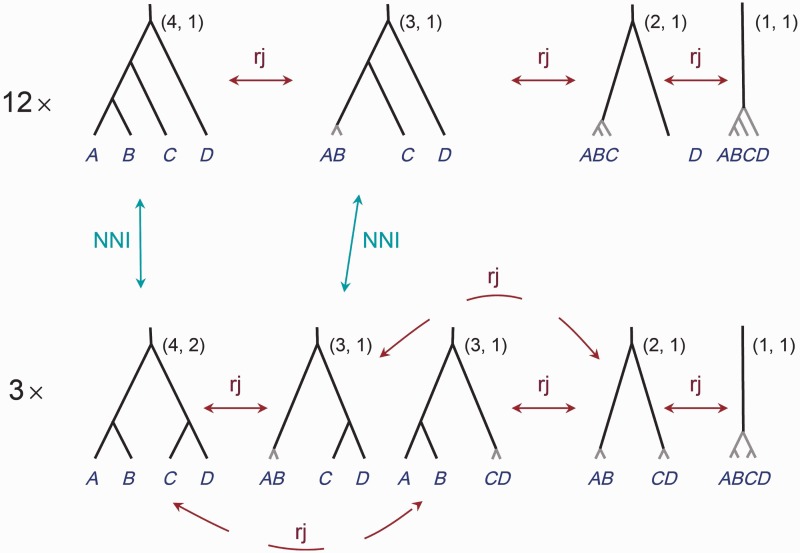
The models of species delimitation and species phylogenies for four populations *A–D*. There should be 15 rows, but only two rows are shown here, to represent the two guide tree shapes. On the same row are the species delimitation models generated by collapsing internal nodes on the same guide tree. The pair of numbers next to each model is the number of species and the number of labeled histories for the species tree. rjMCMC moves between different species-delimitation models are shown, but most of the NNI moves changing species phylogenies are not shown here.

**Fig. 5.**
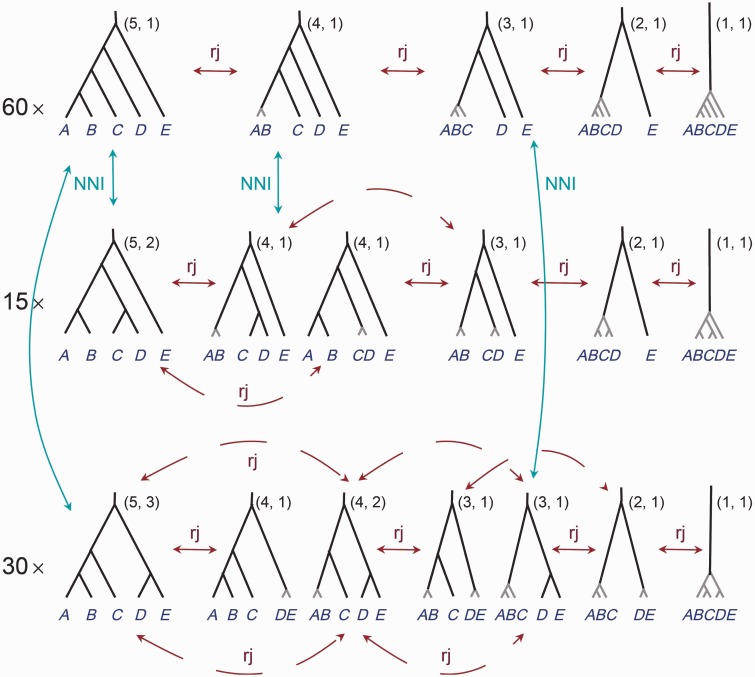
The models of species delimitation and species phylogeny for five populations *A–E*. There should be 105 rows but only three are shown here, to represent the three different guide tree shapes. See legends to figures 3 and 4.

We use NNI to move between species phylogenies with the species delimitation fixed, and rjMCMC to move between species delimitations when the underlying guide tree is fixed. Figure 3 shows all models for the case of three populations as well as the NNI and rjMCMC moves that allow transitions between models. Note that in our formulation, the model of one species has three different representations even though biologically they are equivalent. The case of four populations is illustrated in figure 4, where we show only 2 of the 15 fully resolved species trees (guide trees). The case of five populations is illustrated in figure 5, where we show only 3 of the 105 fully resolved species trees.

We apply the NNI move when there are three or more delimited species in the model. We note that when the number of populations s≥4, NNI applied to models with exactly three delimited species is not sufficient to traverse the space of models. However, NNI applied to models of four delimited species does provide a valid algorithm, as does NNI applied to the fully resolved models (with *s* delimited species). Algorithms applying NNI when there is a particular number of delimited species may be inefficient as it may take a long time for the chain to move from one model to another, and the algorithm may potentially have poor mixing due to difficulties in moving away from local optima in the model space. Such choices may affect the mixing efficiency of the algorithm, but are likely to be data-dependent. In our implementation, we apply NNI as long as there are three or more delimited species in the model. One could also apply NNI to move between different representations of the same model (such as the three representations of the one-species model in fig. 3) when the model has one or two delimited species, although this is not pursued in this study.

### Priors on Species Delimitation and Species Phylogeny

We construct two priors for models of species delimitation and species phylogeny. Prior 0 assigns uniform probabilities to labeled histories (i.e., rooted trees with the internal nodes ranked by age). Prior 1 assigns uniform probabilities to rooted trees. Both priors are specified through a proportional construction.

For the case of three populations, the two priors are equivalent (fig. 3). There are three guide trees, and they have equal probabilities. By collapsing 0, 1, or 2 internal nodes, each guide tree generates three species delimitation models, with 3, 2, and 1 species, respectively (corresponding to each row in fig. 3). These three species delimitation models have equal probabilities. Thus, each of the nine models or representations of figure 3 has probability $\frac{1}{9}$. Note that the model of one single species is represented in three different ways in the algorithm. By summing prior probabilities over the models or representations that have the same number of delimited species, we obtain the prior probabilities for 1, 2, and 3 species to be $\frac{1}{3}$ each.

The case of four populations is illustrated in figure 4. We describe prior 0 first. There are 15 guide trees, with 12 of them having the unbalanced tree shape with one labeled history (fig. 4, first row) and three of them having the balanced tree shape with two labeled histories (fig. 4, second row). Prior 0 assigns uniform probabilities for labeled histories, so that the prior probabilities for the 15 guide trees are proportional to their numbers of labeled histories (1 or 2). By collapsing 0, 1, 2, or 3 internal nodes, the unbalanced guide tree generates four delimitation models, with 4, 3, 2, or 1 species, respectively (and each with one labeled history), and these have equal prior probabilities (fig. 4, first row). Similarly each balanced guide tree generates five delimitation models, with 4, 3, 3, 2, 1 species, respectively, and their probabilities are in proportions 2:1:1:1:1 (fig. 4, second row). The prior probability for any model of species delimitation and species phylogeny can thus be generated. For example, the first model on the second row of figure 4 delimits four species (A,B,C,D), with the phylogeny ((A,B),(C,D)). This has the prior probability $\frac{2}{Z}=\frac{1}{33}$, with Z=12×(1+1+1+1)+3×(2+1+1+1+1)=66 to be the normalizing constant. The second model on the second row of figure 4 delimits three species, with populations A and B grouped into one species, and with the phylogeny to be (AB,(C,D)). This has the prior probability $\frac{1}{Z}=\frac{1}{66}$. By summing prior probabilities over models that have the same number of delimited species, one obtains the prior probabilities for 1, 2, 3, and 4 species to be $P_{1}=P_{2}=\frac{5}{22}=0.2273$, $P_{3}=P_{4}=\frac{6}{22}=0.2727$.

Under Prior 1, with uniform probabilities for rooted trees, the 15 guide trees have equal probabilities $\left(\frac{1}{15}\right)$, and the four or five delimitation models generated by collapsing nodes on the same guide tree also have equal probabilities. Thus, the first two models in the second row of figure 4 have probability $\frac{1}{Z}=\frac{1}{63}$, where *Z* = 63 is the normalizing constant. Overall, the prior probabilities for the number of species are $P_{1}=P_{2}=P_{4}=\frac{5}{21}=0.2381$, $P_{3}=\frac{6}{21}=0.2857$.

The case of five populations is illustrated in figure 5. There are 105 fully resolved guide trees, with 60 of them having the unbalanced shape with one labeled history (fig. 5, first row), 15 having a balanced shape with two labeled histories (fig. 5, second row), and 30 having another balanced shape with three labeled histories (fig. 5, third row). Prior 0 assigns prior probabilities to the fully resolved delimitation models (the guide trees) in proportion to their numbers of labeled histories (1, 2, or 3). By collapsing 0, 1, 2, 3, or 4 internal nodes, the guide tree of the first shape generates five delimitation models, with 5, 4, 3, 2, or 1 species, respectively (fig. 5, first row), and these have equal prior probabilities. The guide tree of the second shape can be collapsed to generate six delimitation models, with 5, 4, 4, 3, 2, 1 species, respectively (fig. 5, second row), and these have probabilities in proportions 2:1:1:1:1:1. The guide tree of the third shape can be collapsed to generate seven delimitation models, with 5, 4, 4, 3, 3, 2, 1 species, respectively (fig. 5, third row), and these have probabilities in proportions 3:1:2:1:1:1:1. By summing over prior probabilities over models of the same number of delimited species, we obtain the prior probabilities for the number of species to be $P_{1}=P_{2}=\frac{7}{47}$ = 0.1489, and $P_{3}=\frac{9}{47}$ = 0.1915, $P_{4}=P_{5}=\frac{12}{47}$ = 0.2553.

Under Prior 1, the 105 guide trees have equal probabilities, and the delimitation models generated by collapsing nodes on the same guide tree also have equal probabilities. Overall, the prior probabilities for the number of species are $P_{1}=P_{2}=\frac{7}{40}=0.175$, $P_{3}=\frac{9}{40}=0.225$, $P_{4}=\frac{10}{40}=0.25$, and $P_{5}=\frac{7}{40}=0.175$.

The two priors are equivalent for three populations, very similar for four populations, and become more different for five populations. With a large number of populations, prior 1 places higher probabilities on models with a small number of delimited species than prior 0. Our analysis of simulated and real data below uses prior 1, which is the default in the BPP program. The prior probabilities for the numbers of delimited species under prior 1 when there are *s* = 3, 4, 5, or 6 populations are summarized in table 1. Under this prior, the probabilities for 1, 2, or *s* delimited species (when the number of populations *s* is fixed) are equal: $P_{1}=P_{2}=P_{s}$, whereas $P_{d}>P_{1}$ for any 3≤d≤s−1.

**Table 1.** Prior Probability for the Number of Delimited Species under Prior 1 (uniform distribution for rooted trees).

Number of Delimited Species	Number of Delimitations	Number of Rooted Trees	Number of Guide Trees	Product	Probability
*s* = 3 populations					
*d* = 1	1	1	3	3	$P_{1}=3/9=1/3=0.333$
*d* = 2	3 (1 2)	1	1	3	$P_{2}=3/9=1/3=0.333$
*d* = 3	1 (1 1 1)	3	1	3	$P_{3}=3/9=1/3=0.333$
*s* = 4 populations					
*d* = 1	1	1	15	15	$P_{1}=15/63=5/21=0.238$
*d* = 2	3 (2 2)	1	1	3	$P_{2}=(3+12)/63=5/21=0.238$
	4 (1 3)	1	3	12	
*d* = 3	6 (1 1 2)	3	1	18	$P_{3}=18/63=6/21=0.286$
*d* = 4	1	15	1	15	$P_{4}=15/63=5/21=0.238$
*s* = 5 populations					
*d* = 1	1	1	105	105	$P_{1}=105/600=7/40=0.175$
*d* = 2	5 (1 4)	1	15	75	$P_{2}=(75+30)/600=7/40=0.175$
	10 (2 3)	1	3	30	
*d* = 3	10 (1 1 3)	3	3	90	$P_{3}=(90+45)/600=9/40=0.225$
	15 (1 2 2)	3	1	45	
*d* = 4	10 (1 1 1 2)	15	1	150	$P_{4}=150/600=10/40=0.250$
*d* = 5	1	105	1	105	$P_{5}=105/600=7/40=0.175$
*s* = 6 populations					
*d* = 1	1	1	945	945	$P_{1}=945/7245=3/23=0.130$
*d* = 2	6 (1 5)	1	105	630	$P_{2}=(630+225+90)/7245$
	15 (2 4)	1	15	225	=3/23=0.130
	10 (3 3)	1	9	90	
*d* = 3	15 (1 1 4)	3	15	675	$P_{3}=(675+540+45)/7245$
	60 (1 2 3)	3	3	540	=4/23=0.174
	15 (2 2 2)	3	1	45	
*d* = 4	20 (1 1 1 3)	15	3	900	$P_{4}=(900+675)/7245$
	45 (1 1 2 2)	15	1	675	=5/23=0.217
*d* = 5	15 (1 1 1 1 2)	105	1	1,575	$P_{5}=1575/7245=5/23=0.217$
*d* = 6	1	945	1	945	$P_{6}=945/7245=3/23=0.130$

Note.—Number of delimitations is the number of ways that *s* populations can be partitioned into *d* delimited species with the given configuration shown in parentheses. The sum over all configurations is the Stirling number of the second kind, *S*(*s*, *d*). For *s* = 5 populations, this is 1, 15, 25, 10, 1 for *d* = 1, 2, 3, 4, 5, respectively; and for *s* = 6, this is 1, 31, 90, 65, 15, 1 for *d* = 1, 2, 3, 4, 5, 6, respectively. The total number of delimitations is given by the sum of *S*(*s*, *d*) over *d*, known as the Bell number. This is 5, 15, 52, 203 for *s* = 3, 4, 5, 6, respectively. Number of rooted trees *R_d_* is the number of rooted tree topologies for *d* species. The total number of models (of species delimitation and species phylogeny) for *s* populations is then given by the product of the number of delimitations and the number of rooted tree topologies, summed over the different configurations. This is 7, 41, 346, 3,797, for *s* = 3, 4, 5, 6, respectively. Number of guide trees is the number of collapsed guided trees that are compatible with the delimitation model; those guide trees correspond to different representations of the same biological model in our algorithm. For example, with *s* = 5 populations, there are S(5,3)=25 possible ways of delimiting three species. Ten of them group three populations into one species with the other two as distinct species (i.e., configuration 1, 1, 3 in the table), such as ABC|D|E. There are three rooted tree topologies for each of such delimitations of *d* = 3 species, and each tree topology, such as ((ABC,D),E), is compatible with three guide trees (which resolve the species *ABC* in different ways) and thus has three representations in our algorithm. Under prior 1, with *s* > 4 populations, $P_{d}>P_{1}=P_{2}=P_{s}$ for 3<d<s−1.

Implementation of prior 1 requires counting the number of labeled histories for a given rooted species tree. This is done as follows. Let *x* and *y* be the number of descendent internal nodes on the left and right part of each internal node. Collapsed internal nodes are treated as tips and are not counted. The number of labeled histories for the given rooted tree is then the product of $\left(\begin{array}{c} x+y\\ x \end{array}\right)$ over the internal nodes.

### Validation of the Theory and Implementation

Our algorithm is complex and extensive testing has been conducted to confirm the correctness of the theory and the implementation. As the likelihood calculation based on sequence alignments was tested extensively before and remains unchanged in this study, our focus has been on the NNI and rjMCMC moves. We ran the program without using sequence data (i.e., by setting the sequence likelihood to 1) to confirm that the MCMC sample matches the prior probabilities for the different models. The prior probabilities for *s* = 3, 4, and 5 populations, described above, have been used for this test. The prior distribution for parameters in the multispecies coalescent model (*θ*s and *τ*s) is confirmed as well.

For even larger numbers of populations, we have convenient predictions for the prior probabilities for all the fully resolved species-tree models (i.e., the guide trees). For prior 0, these probabilities should be proportional to the numbers of labeled histories, whereas for prior 1 they should be uniform. Both priors assign equal probabilities to the different representations of the one-species model in our algorithm (e.g., the 105 representations of the one-species model in the last column of fig. 5 for the case of five populations).

### Summary of the Posterior

The BPP program generates an MCMC sample from the posterior of the models of species delimitation and phylogeny, and the posterior of parameters (*τ*s and *θ*s) under each model. Here, we focus on summaries of the models only. First the model with the highest posterior probability is the maximum a posteriori (MAP) model. We consider biologically equivalent models (such as the three representations of the one-species model of fig. 3 for three populations) to be the same model. The 95% credibility set of models is constructed by collecting the best-supported models until their total posterior probability exceeds 95%. From the posterior distribution of the models, we can calculate the posterior distribution of various summaries, such as the posterior probability of each species delimitation (by ignoring the species phylogeny in the model), the posterior probability of each delimited species, and the posterior probability for the number of delimited species. The MAP estimates of species delimitation and species number can be similarly defined.

## Results

### Simulation Analysis of Statistical Performance

**Fig. 6.**
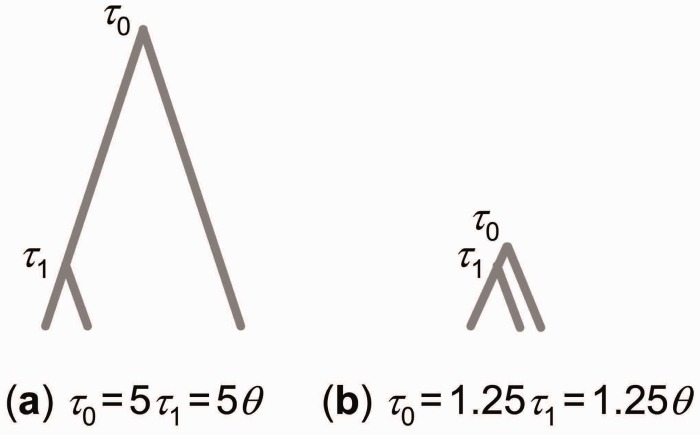
Two species trees used to simulate data, with (*a*) $\tau_{0}=5\tau_{1}$ and (*b*) $\tau_{0}=1.25\tau_{1}$. Data simulated on tree (*a*) will be informative about species phylogeny but not about species delimitation, whereas the opposite is true for data simulated on tree (*b*). The five species on each tree (three contemporary, two ancestral) have the same population size parameter *θ*, and $\tau_{1}=\theta$.

A small simulation study was carried out to examine the influence of the number of loci and the species divergence times on species delimitation probabilities. We considered two combinations of divergence times on trees of three species with either a short or long internal branch length (fig. 6). Sequence data were simulated under the Jukes–Cantor model assuming neutral evolution according to the multispecies coalescent model (Rannala and Yang 2003) using the program MCcoal available in the BPP package. All thecontemporary and ancestral populations had either θ=4Nμ=0.005 or θ=0.001, which correspond to an average of either one substitution (difference) per 200 bases or one substitution per 1,000 bases between a random pair of sequences within each species. Here *N* is the long-term effective population size for the population, and *μ* is the mutation rate per site per generation. We set $\tau_{1}=\theta$ and either $\tau_{0}=5\tau_{1}$ or $\tau_{0}=1.25\tau_{1}$ for θ=0.005, and $\tau_{0}=1.25\tau_{1}$ for θ=0.001. The final combination was explored as a very difficult case, with both small *θ* (limited within population variation) and a very short time duration between species divergence events. We simulated four sequences (two diploid individuals) per species with 1,000 sites per locus and either 1, 2, 5, 10, or 20 loci for a total of 2 × 5 = 10 parameter combinations. For each combination, 50 simulated data sets were generated. BPP was used to analyze the sequence data. For each species two populations were assumed to exist, each comprised one diploid individual (two sequences), with six populations in total. This design allowed the efficiency of BPP in grouping individuals of the same species to be examined. Gamma priors were assigned on parameters. For data simulated using θ=0.005 we used θ∼G(2,400), with mean 0.005 and $\tau_{0}∼G(2,200)$, with mean 0.01. For data simulated using θ=0.001 we used θ∼G(1,1000) and τ∼G(1,1000), both with mean 0.001. Each analysis was conducted twice, using reversible-jump algorithm 0 (with parameter *e* = 2) and algorithm 1 (with parameters *a* = 2 and *m* = 1), respectively (Yang and Rannala 2010). After a burn-in of 10,000 iterations we took 100,000 samples, sampling every two iterations. Results were compared between runs to assess convergence. Additional MCMC runs were performed for particular data sets that appeared to have not converged in the initial runs.

**Fig. 7.**
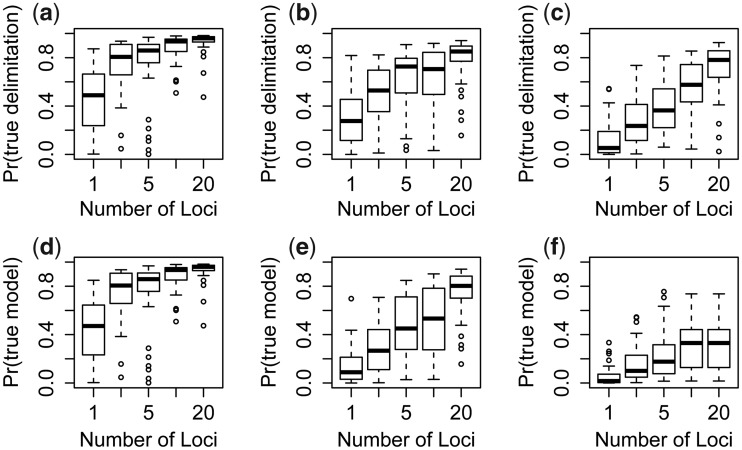
Boxplot of posterior probabilities for the true delimitation (*a*–c) and true model (*d–f*) in data of different numbers of loci simulated with $\tau_{0}=5\tau_{1}$ and $\tau_{1}=\theta =0.005$ (left panels, *a* and *d*), $\tau_{0}=1.25\tau_{1}$ and $\tau_{1}=\theta =0.005$ (middle panels, *b* and *e*), and $\tau_{0}=1.25\tau_{1}$ and $\tau_{1}=\theta =0.001$ (right panels, *c* and *f*). The median is represented by black horizontal lines, the 95% CI by rectangles, the 99% CI by dashed lines, and the outliers as open dots.

We calculated the average MAP model probability over the 50 replicate simulations for each combination of parameters (table 2: Prob). We then calculated the proportion of correct MAP models—those with delimitation and phylogeny matching the ones used to simulate the data (table 2: % Correct). Both the average MAP model probability (a measure of precision) and the proportion of correct MAP models (a measure of accuracy) increase with the number of loci and with the increase of *τ*_0_. In all but one case, the percentage of correct models is greater than the average MAP model probability (the exception is for data of one locus simulated with θ=0.001 in which case the method favors a single species); these results support the notion that Bayesian methods often have good Frequentist properties and that a high posterior probability corresponds to at least as high a proportion of correct models. The distributions of posterior probabilities for the true model and true delimitation are shown in figure 7 as a function of the number of loci for data simulated with either a large *τ*_0_ (=5θ) or small *τ*_0_ (=1.25θ) and θ=0.005 or θ=0.001. Note that for the model to be correct, the species delimitation has to be correct (and in addition the species phylogeny has to be correct), so that Pr{ true model }≤Pr{ true delimitation }. The two probabilities are nearly the same (fig. 7*a* and *d*) for the species tree model of figure 6*a*, whereas they are more different (fig. 7*c* and *f*) for the species tree model of figure 6*b* with θ=0.001. This is because in the latter case, there are errors in the species phylogeny even when the species are correctly delimited.

**Table 2.** Average MAP Probability of Model versus Percent Correct.

Number of Loci	$\theta =0.005,\tau_{0}=5\times \theta$		$\theta =0.005,\tau_{0}=1.25\times \theta$		$\theta =0.001,\tau_{0}=1.25\times \theta$	
	Prob	% Correct	Prob	% Correct	Prob	% Correct
1	0.53	0.64	0.28	0.30	0.17	0.06
2	0.77	0.92	0.40	0.52	0.24	0.32
5	0.83	0.88	0.53	0.76	0.34	0.42
10	0.89	1.0	0.61	0.70	0.39	0.52
20	0.93	1.0	0.78	0.94	0.57	0.78

Note.—Prob is the average probability of the MAP model over all the simulated data sets for each specific combination of simulation parameters and % correct is the proportion of these data sets for which the delimitation and phylogeny both matched the true model used in the simulation (i.e., the MAP model is the true model).

### Analysis of Two Empirical Data Sets

#### The Coast Horned Lizard Data

**Fig. 8.**
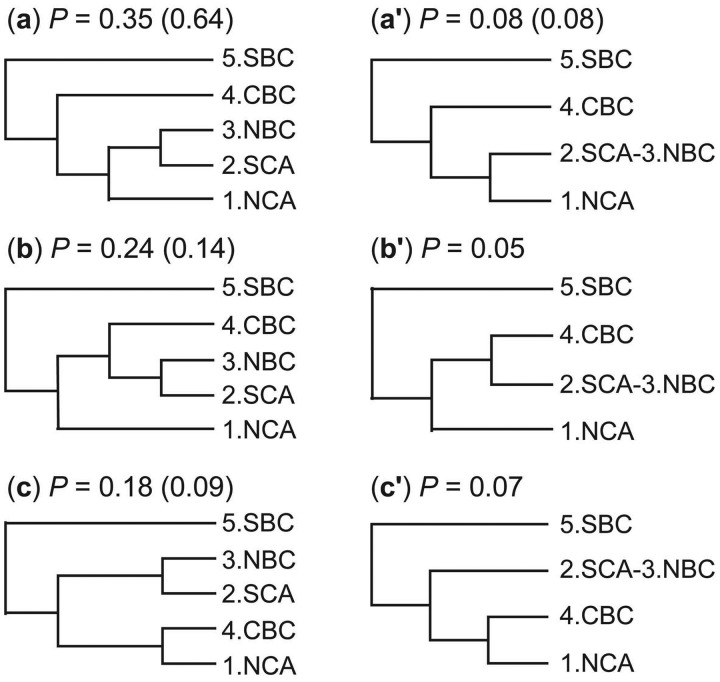
Posterior probabilities for six models of species delimitation and species phylogeny in the 95% credibility set for the coast horned lizard data, obtained from a BPP analysis under the prior $\tau_{0}∼G(2,1,000)$ and θ∼G(2,100). Models (*a*′)–(*c*′) are identical to (*a*)–(*c*), respectively, except that 2.SCA and 3.NBC are one species. Note that species trees of (*a*), (*b*), (*a*′), and (*b*′) are consistent with the geographical distributions of the populations, but those of (*c*) and (*c*′) are not. The posterior probabilities in parentheses are for the top four models under the prior $\tau_{0}∼G(2,1000)$ and θ∼G(2,1000).

The first data set we analyze includes two nuclear loci (*BDNF*: 132 sequences, 529 bp; and *RAG-1*: 136 sequences, 1,100 bp) sampled from coast horned lizards originally published by Leaché et al. (2009) and previously reanalyzed by Rannala and Yang (2013). Assignment is based on an mtDNA phylogeny, with five phylogeographic groups arranged latitudinally: North California (1.NCA), South California (2.SCA), Northern Baja California (3.NBC), Central Baja California (4.CBC), and South Baja California (5.SBC) (see fig. 8). There are thus five populations in the BPP analysis. We use the same priors as in Rannala and Yang (2013): $\tau_{0}∼G(2,1000)$ for the root of the species tree and θ∼G(2,100). After a burn-in of 4,000 iterations, we took $2\times 10^{5}$ samples, sampling every four iterations. Multiple runs using both rjMCMC algorithms 0 and 1 were used to ensure consistency between runs. Each run took about 9 h.

The six models of species delimitation and species phylogeny with the highest posterior probabilities are shown in figure 8. These have either five species or four species. In the latter case, populations 2.SCA and 3.NBC belong to the same species. These six models constitute the 95% credible interval (CI) of models, with a total probability of *P* = 0.97. The 99% CI includes two additional models, each of five species, with a total probability of *P* = 0.997. Populations 2.SCA and 3.NBC have the probability 0.20 of being the same species. All other populations are distinct species with probability 1. The probability for five species is 0.80 and that for four species is 0.20. The fixed guide tree used in Rannala and Yang (2013) is incompatible with all six models of figure 8, and use of that guide tree generated the posterior probability 1.00 for five species. By allowing phylogenetic uncertainties, the method provided weaker support for five delimited species in the present analysis.

The prior θ∼G(2,100), with mean 0.02, appears to be a poor choice as the sequences in the data set are highly similar. We thus repeated the analysis using the prior θ∼G(2, 1000), with mean 0.002. Parameter *τ*_0_ has the same prior *G*(2, 1000) as before. With this prior, the analysis favors largely the same models, but with more extreme posterior probabilities. The top four models of species delimitation and species phylogeny are shown in figure 8. They collectively have the posterior probability 94.6%. The posterior probability for five delimited species is 90%, compared with the 80% under the θ∼G(2,100) prior. The posterior probability for four delimited species (with 2.SCA and 3.NBC grouped into one species) is 10%. The analysis suggests considerable impact of the prior on *θ*, with large *θ*s favoring fewer species.

We used the prior θ∼G(2,1000) and $\tau_{0}∼G(2,1000)$ to estimate the parameters under the multispecies coalescent model with the species tree fixed at the MAP tree (fig. 8*a*). The posterior mean and the 95% CI for *τ*_0_ for the root are 0.0012 (0.00056, 0.0018). The posterior means of the nine *θ* parameters are in the range 0.0009–0.0066.

#### The Cavefish Data

The second data set we analyze consists of five nuclear gene loci: *s7, rag1, myh6, plagl2*, and *tbr1*, sampled from 22 individuals of the species complex (*Typhlichthys subterraneus*) (Niemiller et al. 2012), with one sequence for each individual at each locus. *Typhlichthys subterraneus* is a teleost fish widely distributed in Eastern North America. Because of convergent evolution, species delimitation based on morphology is difficult and may miss cryptic species. The individuals are assigned to six populations (*A*–*F*) identified by Niemiller et al. (2012). Two sequences from the outgroup species *Speoplatyrhinus poulsoni* (Sp) are also included in the data at each locus. The priors used are $\tau_{0}∼G(2,1000)$ and θ∼G(2,1000). Both have a mean of one mutation per kilobase. After a burn-in of 4,000 iterations, we take $2\times 10^{5}$ samples, sampling every four iterations. Each run took about 1.5 h.

The posterior probability distribution of the models is rather diffuse. The MAP model is (*Sp*, ((*C*, *D*), (*F*, (*B*, (*A*, *E*))))), with seven species and *P* = 0.15. The next most probable model has seven species as well, with a slightly different phylogeny and *P* = 0.11. The third most probable model has six species, with *C* and *D* grouped into one species and *P* = 0.08. The 95% (or 99%) credibility set of models includes as many as ∼80 (or ∼180) distinct models. Averaged over all models, the posterior is 0.69 for seven species, 0.30 for six species (0.27 for grouping *C* and *D* into one species, and 0.03 for grouping *A* and *E* together), and 0.01 for five species (with *C* and *D* grouped in one species and *A* and *E* grouped in another). Populations *B* and *F* are distinct species with posterior probability 1. By using the fixed guide tree (*Sp*, ((*C*, *D*), ((*B*, *F*), (*A*, *E*)))), Rannala and Yang (2013) obtained the posterior *P* = 0.60 for seven species. This (fully resolved) seven-species model is the best among those that are compatible with that guide tree, and ranks only sixth in the present analysis, with *P* = 0.04. Overall there is a lot of uncertainty in the posterior, especially concerning the species phylogeny.

We also used the same priors on *θ* and *τ*_0_ to estimate the parameters in the multispecies coalescent model with the species tree fixed at the MAP tree: (*Sp*, ((*C*, *D*), (*F*, (*B*, (*A*, *E*))))). The posterior mean and the 95% CI for *τ*_0_ for the root are 0.0048 (0.0033, 0.0065). The posterior means of the *θ* parameters range from 0.0011 to 0.0132.

## Discussion

### The Impact of Priors on Bayesian Species Delimitation

In this study, a fully specified model defines both the species delimitation and the species phylogeny. It defines the parameters (*θ*s and *τ*s in the multispecies coalescent), specifies the gene tree distributions, and defines the likelihood function. From table 1, the number of delimitations is 5, 15, 52, 203, for *s* = 3, 4, 5, 6 populations, respectively, whereas the number of models is 7, 41, 346, 3,797, for *s* = 3, 4, 5, 6, respectively. Note that a delimitation may not be a fully specified model: Knowledge of the delimitation (without the knowledge of the species phylogeny) may not be sufficient to define the parameters or to specify the probabilistic distributions of the gene trees. For example, in the case of *s* = 5 populations (A,B,C,D,E), the delimitation ABC|D|E (for *d* = 3 species) is not a fully specified model as it is insufficient to specify the gene-tree distributions or the likelihood function. However, the species phylogeny, ((ABC,D),E) is a fully specified model.

Bayesian model comparison requires specification of prior probabilities on the (fully specified) models. Suppose model *i* has prior probability *π_i_* and marginal likelihood *L_i_*. Then, the posterior probability for model *i* is given as

(1) $${\mathbb{P}}_{i}\propto \pi_{i}L_{i},$$

where the proportionality constant ensures that the posterior probabilities for all models sum to 1. Note that the marginal likelihood *L_i_* should be calculated by integrating over the parameters *θ*s and *τ*s in the multispecies coalescent (and by averaging over the gene trees at all loci).

Given the great number of models under comparison and the intricate relationships among them, specifying the prior for models (*π_i_*) is not an easy task. One possible prior is to assign uniform probabilities for all models. For *s* = 5 populations, this assigns the prior probability 1/346 for one species and 105/346 for five species. Another prior assigns uniform probabilities for all species delimitations and then divides the probability for each species delimitation uniformly among the species phylogenies given the delimitation. For *s* = 5 populations, this assigns the prior probability 1/52 for one species and 1/52 for five species. We suggest that for most biological situations, neither the uniform prior for the delimitations nor the uniform prior for the models is sensible, as both favor many delimited species, especially if a large number of populations exist in the analysis (table 1). These two priors are not implemented in BPP. In contrast, both priors 0 and 1, discussed in this article and implemented in BPP, favor fewer species for a given number of populations (*s*).

Another possibility is to use the Dirichlet process to partition the populations into delimited species. This prior has the drawback that the number of delimited species grows fairly quickly with the number of populations. The Dirichlet process also has the property of “rich getting richer,” favoring partitions (delimitations) that are highly unbalanced, with a few large partitions and many very small partitions (Green and Richardson 2001). This may not be a desirable feature. Two other interesting priors may be suitable when a large number of populations exist in the analysis. These assign uniform probabilities for the number of delimited species (1, 2, . . ., *s*). The prior probability ($\frac{1}{s}$) for each number of delimited species is then divided up among the compatible models (of species delimitation and species phylogeny) either uniformly or in proportion to the labeled histories, in the same way that priors 0 and 1 are constructed.

Note that because of equation (1), the prior probabilities for models have a direct impact on their posterior probabilities. If the number of populations (*s*) is large, the different prior specifications may induce very different prior probabilities for the number of delimited species. Note also that in theory the posterior probabilities of models under one prior can be converted into the posterior probabilities of models under another. Consider two priors *π* and π′. The first assigns prior probabilities *π_i_* and *π_j_* for two models *i* and *j*, whereas the second assigns $\pi \prime_{i}$ and $\pi \prime_{j}$. Suppose the posterior probabilities for the two models under the prior *π* are calculated to be ${\mathbb{P}}_{i}$ and ${\mathbb{P}}_{j}$. Then from equation (1), we have the posterior probabilities for the two models under the prior π′ to be given as

(2) $$\frac{\mathbb{P}\prime_{i}}{\mathbb{P}\prime_{j}}=\frac{\pi \prime_{i}}{\pi \prime_{j}}\times \frac{L_{i}}{L_{j}}=\frac{\pi \prime_{i}}{\pi \prime_{j}}\times \frac{\pi_{j}}{\pi_{i}}\times \frac{{\mathbb{P}}_{i}}{{\mathbb{P}}_{j}}.$$

Here, the marginal likelihood ratio $\frac{L_{i}}{L_{j}}$ is the Bayes factor for comparing models *i* and *j*.

Besides the prior probabilities on the models, the prior on parameters in each model may also affect posterior model comparison. In particular, we observed in both simulated and real data sets that the prior on *θ*s may have considerable effects on posterior probabilities of models of species delimitation. A very large *θ* makes it possible for delimitation models of fewer species to fit the data and thus a large prior mean on *θ* tends to favor fewer species (Zhang et al. 2011).

### Inference of Assignment

Our algorithm attempts to group different populations into one species and also explores different phylogenetic relationships among the delimited species. It does not attempt to split any population into different species. If one assigns every individual in the data sample into a different population (as in our simulation study), the algorithm will infer assignments, species delimitation, and species phylogeny in one joint analysis. Although this is in theory possible and the results of our limited simulations suggest that the BPP program can be accurate in assigning individuals and delimiting species, we envisage at least two difficulties with such an analysis. First, the analysis is feasible computationally for relatively small data sets only and may not be practical for a large sample. The computation increases far more quickly with an increase in the number of populations than with an increase in the number of sequences at each locus. Second, both priors 0 and 1 described in this article and implemented in BPP may be inappropriate for such an analysis if many individuals are sampled, as they favor a large number of delimited species (table 1). If we want to determine whether certain geographical populations are distinct species and sample increasingly more individuals from each geographical population, we should not a priori expect the number of species to increase when more samples are collected from each geographical population.

We envisage that often strong evidence (for instance, based on morphological and behavioral differences or geographic distributions) may be available to decide that certain individuals should belong to the same population or species (Olave et al. 2014). We therefore recommend the use of such information to assign individuals to populations to reduce the state space for the MCMC algorithm and to reduce the impact of the prior.

### Software Availability

The algorithms described in this article are implemented in the program BPP Version 3, which may be downloaded from http://abacus.gene.ucl.ac.uk/software/ (last accessed October 14, 2014). The program documentation and sequence data for the cavefish and coast horned lizard examples presented in this article are included in the program package.
